# Delayed surveillance colonoscopy after piecemeal EMR is not associated with increased recurrence rates

**DOI:** 10.1055/a-2816-4998

**Published:** 2026-03-06

**Authors:** Tarek Arraf, Yuri Gorelik, Fares Mazzawi, Halim Awadie, Suzan Banna, Rawia Moalem, Nour Ershaid, Rashad Falah, Andrew Beany, Itay Maza, Amir Klein

**Affiliations:** 1 58878 Gastroenterology, Department of Internal Medicine D, Rambam Health Care Campus, Haifa, Israel; 2 58880 The Ruth and Bruce Rappaport Faculty of Medicine, Technion Israel Institute of Technology, Haifa, Israel; 3 61172 Gastroenterology Institute, Ha'emek Hospital, Afula, Israel; 4 Gastroenterology, The Holy Family Hospital Nazareth, Nazareth, Israel

**Keywords:** Endoscopy Lower GI Tract, Polyps / adenomas / ..., Endoscopic resection (polypectomy, ESD, EMRc, ...), Quality and logistical aspects, Quality management

## Abstract

**Background and study aims:**

First surveillance colonoscopy (SC1) following piecemeal endoscopic mucosal resection (pEMR) is recommended at 3 to 6 months. We aimed to investigate whether delayed SC1 is associated with increased risk of recurrence.

**Patients and methods:**

This was a retrospective analysis of a prospective cohort of patients undergoing pEMR for large non-pedunculated colorectal polyps (LNPCPs). Patients were categorized into standard SC1 (3–6 months) and delayed SC1 (> 6 months) groups. The primary endpoint was recurrence at SC1. Secondary outcomes included recurrence at second surveillance colonoscopy (SC2) and features of lesion recurrence. Subgroup analyses of very delayed SC1 and high-risk lesions were performed. Recurrence rates were also evaluated with propensity score matching (PSM).

**Results:**

We analyzed 577 lesions (standard: 407, delayed: 170) with a median polyp size of 30 mm (interquatile range [IQR] 25–40). Median time to SC1 was 5 months (IQR 5–6) in the standard versus 9 months (IQR 7–13) in the delayed group ( *P* < 0.01). There were no significant differences in SC1 recurrence rates between groups (6.4% versus 6.5% in the standard and delayed respectively, *P* = 1). At SC2, recurrence rate was also similar between groups (3.8% versus 8.8% respectively, *P* = 0.43). Recurrence rates did not differ significantly among the analyzed subgroups. Recurrence rates did not differ significantly among the analyzed subgroups or in the PSM analyses. No advanced recurrences were detected at SC1 or SC2.

**Conclusions:**

Delayed SC1 is not associated with increased recurrence rates. Complete resection can be anticipated for most lesions and SC1 at 1-year post resection may be sufficient.

## Introduction

Colorectal cancer (CRC) is one of the most prevalent cancers worldwide with significant morbidity and mortality 1 . Numerous studies have shown that removal of colorectal polyps reduce incidence and mortality of CRC 2 . Large non-pedunculated colorectal polyps (LNPCPs) are considered high-risk precursors of colorectal cancer. Piecemeal endoscopic mucosal resection (pEMR) is recommended as the treatment of choice for most noninvasive LNPCPs by societal guidelines because it offers an excellent safety and efficacy profile 3 4 5 6 .

One of the major drawbacks of colonic pEMR is residual and recurrent adenoma (RRA). Previous studies showed that RRA can occur in 15% to 30% of cases. Risk factors for recurrence include high-grade dysplasia, lesion size, lesion location, intra-procedural bleeding, and previous attempt 7 8 9 . Recurrence at first postpolypectomy surveillance colonoscopy (SC1) can be identified endoscopically with very high accuracy 10 and endoscopic treatment during SC1 with re-excision of the RRA with or without ablation 11 results in a very low residual RRA at second postpolypectomy surveillance colonoscopy (SC2) of 4% 7 .

Current data show that RRA rates are significantly reduced to the range of 3% to 5% with application of ablative measures such as snare-tip soft coagulation (STSC) or Argon plasma coagulation to the post EMR resection margin in a systematic fashion 6 7 8 12 13 .

Current surveillance schedule following pEMR includes SC1 at 3 to 6 months after the index procedure and SC2 1 year after SC1. This results in additional burden on the patients and the health care system and may hinder patient compliance 9 . It is plausible that when RRA at SC1 is extremely low, the effect of the first (early) surveillance on long-term recurrence rates will be negligible.

This study aimed to investigate whether delayed SC1 is associated with an increased risk of recurrence.

## Patients and methods

### Study design

This was a retrospective analysis of a prospectively collected cohort of adult patients referred for standard pEMR of LNPCPs (≥ 20) at two academic centers in Israel (Rambam Health Care Campus, and Nazareth Holy Family Hospital) from March 2016 to December 2024. SC1 was recommended 3 to 6 months after the index procedure in all cases; delayed SC1 reflected real-world deviations (eg, missed appointments/non-adherence, scheduling constraints, COVID-19-related disruptions, and occasional miscommunication). Reasons for delay were not captured in a fully structured manner for every case.

We compared two groups according to their SC1 time. The standard group was composed of patients who completed SC1 within 3 to 6 months, whereas the delayed group consisted of patients who completed SC1 after 6 months or more.

### Patients

We included all patients who underwent pEMR of LPCPs and completed SC1 with available data on SC1 findings.

### Ethics statement

All patients gave informed consent and the study was approved by the hospitals review board (RMC-0197–18, P94523).

### Data collection

We collected pre-procedure demographic patient information, lesion location, procedure data, and data on surveillance colonoscopies, recurrences and treatment performed during SC1.

### Procedure

All lesions in this cohort were LNPCPs (> 20 mm) and all resections were performed by piecemeal EMR (no en bloc resections). All procedures and surveillance colonoscopies were performed by four expert endoscopists with dedicated advanced endoscopy/EMR fellowship training, who routinely performed pEMR with systematic margin ablation. We used high-definition scopes (Olympus 190 series Q190 PCF/CF; Olympus, Tokyo, Japan or Fuji 760 series). All LNPCPs underwent inspection and photo documentation with high-definition white light and optical chromoendoscopy prior to resection to exclude features suggestive of deep submucosal invasion. In both centers, standard pEMR technique was used with the objective of complete snare excision 14 . Submucosal injections were performed with either succinilated gelatine (Gelofusin) or normal saline. Snare size and type were chosen at the discretion of the endoscopist performing the EMR. Mostly 15- or 20-mm braided snares were preferred.

After complete resection, standard post resection inspection of the mucosal defect to exclude residual adenoma and deep injury was performed 10 . This was followed by thermal ablation of the entire mucosal defect margin with snare soft tip coagulation (STSC; SOFT COAG, 80 W Effect 4; ERBE Electromedizin, Tubingen, Germany), creating a 2- to 3-mm rim of completely ablated tissue 13 . In all cases, SC1 was recommended 3 to 6 months after the index procedure as per societal guidelines 6 15 . Scar evaluation at surveillance was performed with high-definition white light and virtual chromoendoscopy. In both centers, routine biopsies were taken from scars in the first 100 cases. We compared histology with the endoscopic assessment and confirmed a negative predictive value of 99% for correctly identifying a normal scar without recurrence. Afterward, we relied on endoscopic diagnosis for recurrence and biopsies were taken only when recurrence was suspected endoscopically. This approach has been previously validated 16 .

The database did not systematically capture whether piecemeal resection was planned pre-procedurally or converted intra-procedurally, or the specific reasons for conversion (eg, non-lifting, fibrosis, difficult location).

All recurrences were treated with either cold snare, Cold-forceps avulsion with adjuvant STSC (CAST).

### Histopathology

All resected fragments from each lesion were retrieved and submitted in formalin for histopathological evaluation at each center. Specimens were reviewed by gastrointestinal pathologists using standard criteria. When advanced pathology was identified (high-grade dysplasia or carcinoma), the diagnosis was independently confirmed by a second gastrointestinal pathologist to finalize diagnosis and management.

### Study endpoints

Our primary endpoint was the rate of recurrence at SC1. Secondary outcomes included, recurrence rates at SC2, subanalysis of high-risk lesions for recurrence, and presence of advanced pathology (high-grade dysplasia or cancer) in SC1 and SC2 recurrences.

### Statistical analysis

All statistical analysis was performed per lesion. Descriptive statistics were presented as means with standard deviations (SDs) for continuous data and as absolute numbers with percentages for categorical data. Baseline characteristics and outcomes were compared between the standard and delayed SC1 groups. The Student's *t* -test was employed for comparing continuous variables, whereas chi-square tests were used for categorical variables. Univariate analysis was performed to identify risk factors for recurrence.

To plot the adjusted rate of recurrence as a function of months to SC1 we used a multivariable logistic regression model with endoscopic recurrence as outcome and the predictors—months to SC1, lesion size, and high-risk lesion status. Predicted probabilities and their 95% confidence intervals (CIs) were obtained by back-transforming the model’s logit estimates, and these estimates were visualized over a continuum of months to SC1.

Propensity score matching (PSM) was performed to create balanced cohorts of patients with standard versus delayed SC1 by matching the factors gender and lesion size, which are associated with risk of recurrence and were unbalanced in the cohort. Matching was performed using a nearest neighbor algorithm with a 1:1 ratio, applying tight calipers (0.2 SD) and excluding observations outside the common support. Baseline covariate balance in the matched sample was subsequently assessed using paired statistical tests—Wilcoxon signed rank for continuous variables and McNemar’s tests for categorical variables.

Several subgroup analyses were performed. We compared patients in the standard group with patients who underwent SC1 12 months or more after the pEMR. We also evaluated separately only patients with high-risk lesions and patients without high-risk lesions, defined as having one or more of the following features: full circumferential lumen extension, size larger than 80 mm, ileocecal valve involvement, anorectal junction involvement, or previously attempted resections.

All tests were two-tailed and *P* < 0.05 was considered statistically significant. Statistical analyses were executed using R software (version 4.2.0; the R Foundation for Statistical Computing).

## Results

### Baseline characteristics

**Fig. 1:**
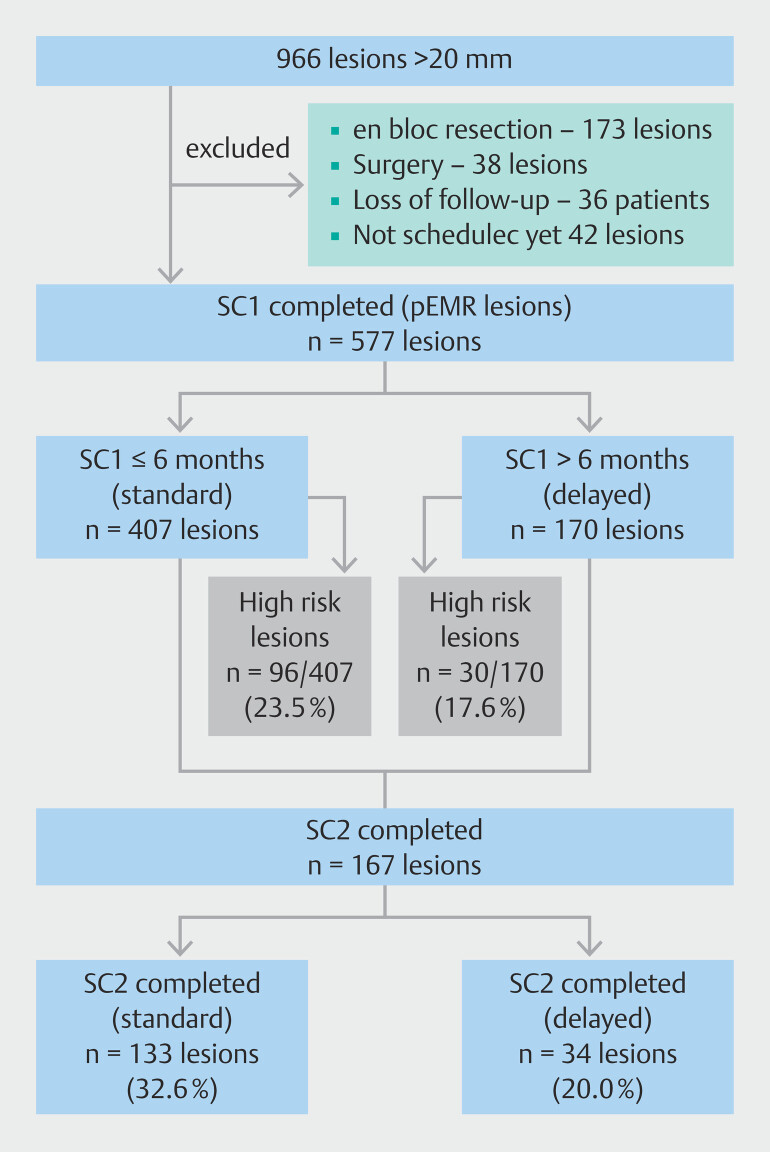
Flowchart.

The study flowchart is presented in Fig. 1 . Complete data were available for 577 lesions taken from 497 patients who completed SC1. A total of 407 and 170 lesions were included in the standard and delayed groups respectively. Median time to SC1 was 5 months (interquartile range [IQR] 5.0–6.0) in the standard versus 9 months (IQR 7.0–13.0) in the delayed group ( *P* < 0.01).

Baseline characteristics of the lesions in each group are presented in Table 1 . Both groups demonstrated comparable age distributions, with a median age of 66 years (IQR 59.0–72.7) in the standard group and 68 years (IQR 59.0–75.0) ( *P* = 0.06) in the delayed SC1 group. A higher percentage of female patients was observed in the standard group (46.6% versus 33.5%, *P* = 0.01).

**Table:** **Table 1** Baseline characteristics of the cohort.

	Standard group (n = 407)	Delayed group (n = 170)	*P* value
Age, years (median [IQR])	66.0 [59.0–72.7]	68.0 [59.0–75.0]	0.06
Female, n (%)	189 (46.6)	57 (33.5)	0.01
Polyp size, mm (median [IQR])	35.0 [25.0–40.0]	30.0 [25.0–40.0]	0.01
Time to SC1, months (median [IQR])	5.0 [5.0–6.0]	9.0 [7.0–3.0]	< 0.01
Polyp location, n (%)			0.25
Anorectal	63 (15.5)	17 (10.0)	
Sigmoid colon	37 (9.1)	14 (8.2)	
Descending colon	25 (6.1)	10 (5.9)	
Transverse colon	76 (18.7)	39 (22.9)	
Ascending colon	108 (26.5)	39 (22.9)	
Cecum	85 (20.9)	40 (23.5)	
Ileocecal valve	13 (3.2)	11 (6.5)	
High-grade dysplasia at Index Colonoscopy, n (%)	101 (24.8)	36 (21.2)	0.41
STSC, n (%)	392 (96.6)	162 (95.3)	0.63
Intra-procedural bleeding, n (%)	9 (11.0)	3 (10.0)	1
High-risk lesion, n (%)	96 (23.6)	30 (17.6)	0.14
SC2 completed. n (%)	133 (32.7)	34 (20.0)	< 0.01
IQR, interquartile range; SC1, first surveillance colonoscopy; SC2, second surveillance colonoscopy; STSC, snare tip soft coagulation.			

Polyp sizes were larger in the standard group versus the delayed group (median size 35 mm (IQR 25.0–40.0) versus 30 mm (IQR 25.0–40.0, *P* = 0.01). Distribution of polyps along the colon also varied slightly between the groups, with a higher occurrence of lesions in the anorectal region in the standard group and a higher percentage of lesions in the ileocecal valve in the delayed group ( *P* = 0.25; Table 1 ). No significant difference between the groups was observed in the rate of high-grade dysplasia (24.8% in the standard group versus 21.2% in the delayed group, *P* = 0.41), or in intra-procedural bleeding (11.0% versus 10.0%, *P* = 1).

### Outcomes

Comparison of outcomes between groups is presented in Table 2 . Both groups had similar recurrence rates at SC1 (6.4% standard group versus 6.5% delayed group, *P* = 1). Data on SC2 were available for 167 of 577 lesions overall (28.9%) (133 lesions in the standard group and 34 lesions in the delayed group). There was no statistically significant difference in recurrence rate at SC2 between the two groups (3.8% standard group versus 8.8% the delayed group; *P* = 0.43).

**Table:** **Table 2** Primary and secondary outcomes.

	Standard group	Delayed group	*P* value
Endoscopic recurrence at SC1, n (%)	26 (6.4)	11 (6.5)	1
*Advanced pathology at SC1	0	0	1
Endoscopic recurrence at SC2, n/N (%)	5 (3.8)	3 (8.8)	0.43
High-risk lesions recurrence rate, n (%)	11 (11.5)	4 (13.3)	1
Without high-risk lesions recurrence rate, n (%)	15 (4.8)	7 (5.0)	1
Advanced pathology is high-grade dysplasia/cancer. SC1, first surveillance colonoscopy; SC2, second surveillance colonoscopy. Note: SC2 recurrence percentages are calculated among lesions that completed SC2 (standard N = 133; delayed N = 34).			

Among lesions with SC2 completed (n = 167), recurrence at SC2 was observed in eight lesions (4.8%). Of these, three of 167 (1.8%) were SC1-negative and became SC2-positive, and five of 167 (3.0%) were SC1-positive at SC1, treated endoscopically, and were SC2-positive. By surveillance group, SC2 recurrence occurred in five of 133 lesions (3.8%) in the standard group and three of 34 lesions (8.8%) in the delayed group; the SC1-negative to SC2-positive cases occurred in the standard group, whereas all delayed-group SC2 recurrences occurred after SC1 recurrence and treatment. A factual breakdown is provided in Supplementary Table 1.

We conducted a subgroup analysis comparing the standard group versus patients who had very late SC1 (> 12 months after the pEMR, median time to SC1 16 months (IQR 13.0–19.0). This group contained 53 lesions. No significant differences in recurrence rates were seen in these groups (6.4% in the standard versus 7.5% in the very late group, *P* = 0.98, Table 3 ). Importantly, we found no cases of advanced neoplasia or cancer during SC1 or SC2 in either group.

**Table:** **Table 3** Standard group vs very late group (≥ 12 months).

	Standard group (n = 407)	Very delayed group (n = 53)	*P* value
Endoscopic recurrence at SC1, n (%)	26 (6.4)	4 (7.5)	0.98
Advanced pathology at SC1	0	0	1
SC2 completed, n (%)	133 (32.7)	6 (11.3)	< 0.01
Endoscopic recurrence at SC2, n/N (%)	5 (3.8)	2 (33.3)	0.02
Advanced pathology refers to high-grade dysplasia/cancer. SC1, first surveillance colonoscopy; SC2, second surveillance colonoscopy. Note: SC2 recurrence percentages are calculated among lesions that completed SC2 (standard N = 133; very delayed N = 6).			

We performed an additional subgroup analysis comparing the standard and delayed SC1 in high-risk lesions (> 80 mm; n = 16), ileocecal valve involvement (n = 24), anorectal junction involvement (n = 80), or previously attempted lesions (n = 6). No significant differences were seen in recurrence rates in the groups in these lesions although the absolute values for both groups were significantly higher (11.5% in the standard group versus 13.3% in the delayed group, *P* = 1).

In the 311 lesions without high-risk features in the standard group and 140 non high-risk lesions in the delayed group, the recurrence rate at SC1 was also similar between these groups (4.8% in the standard group versus 5.0% in the delayed group; *P* = 1).

PSM analysis matched for gender and polyp size matched 169 lesions in the delayed group to 169 lesions in the standard group. Matching parameters and additional baseline features were balanced between groups ( Table 4 ). Recurrence rates were similar between the matched groups (7.1% in the standard group versus 6.5% in the delayed group, *P* = 1, Table 4 ).

**Table:** **Table 4** Propensity score matching analysis for gender and polyp size.

	Standard group (n = 169)	Delayed group (n = 169)	*P* value
Age, years (median [IQR])	66.30 [60.0–72.70]	68.0 [59.0–75.0]	0.118
Female, n (%)	55 (32.5)	57 (33.7)	0.908
Polyp size, mm (median [IQR])	30.0 [25.0–40,0]	30.0 [25.0–40.0]	0.727
Time to SC1, months (median [IQR])	5.0 [5.0–6.0]	9.0 [7.0–13.0]	< 0.001
High-grade dysplasia at index colonoscopy, n (%)	39 (23.1)	36 (21.3)	0.793
High-risk lesion, n (%)	45 (26.6)	30 (17.8)	0.067
Endoscopic recurrence at SC1, n (%)	12 (7.1)	11 (6.5)	1
SC2 completed. n (%)	63 (37.3)	34 (20.1)	0.001
Endoscopic recurrence at SC2, n/N (%)	4 (6.3)	3 (8.8)	0.97
Advanced pathology refers to high-grade dysplasia/cancer. IQR, interquartile range; SC1, first surveillance colonoscopy; SC2, second surveillance colonoscopy. Note: In the propensity score matched cohort, SC2 recurrence percentages are calculated among lesions that completed SC2 (standard N = 63; delayed N = 34).			

**Fig. 2:**
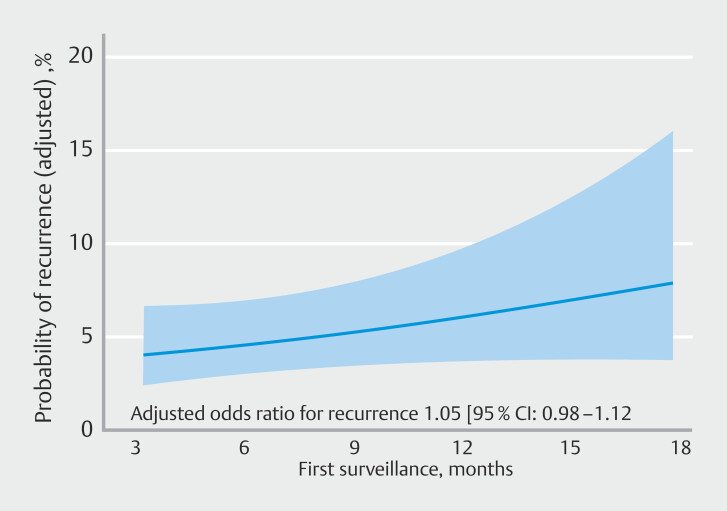
Adjusted probability of recurrence plotted during time in months.

The adjusted association between time to SC1 and predicted probability of endoscopic recurrence is presented in Fig. 2 . As demonstrated interval to SC1 was not significantly associated with probability of recurrence (adjusted odds ratio [OR] 1.05; 95% CI 0.98–1.12).

## Discussion

The recommended timing for surveillance colonoscopies following pEMR of LNPCPs is currently based on older data which anticipated recurrence rates of up to 20% to 30% during SC1 6 7 8 9 17 . This is why early surveillance (between 3–6 months after the index procedure) was recommended to avoid large recurrences that may contain advanced neoplasia, and pose a significant challenge for complete and curative endoscopic resection. However, recent advances in EMR technique have resulted in a significant decrease in polyp recurrence at SC1 following piecemeal resection of LNPCPs. We hypothesize that with these new capabilities, early surveillance may be avoided becausse risk of early recurrence is extremely low. In fact, it is very close to recurrence rates observed after colonic ESD, where first surveillance is recommended at 1 year after the index procedure 18 . Therefore, we sought to explore the effect of late SC1 on recurrence rates in a large EMR database.

Our standard surveillance group completed SC1 with a median time to SC1 of 5 months (IQR 5.0–6.0) compared with 9 months (IQR 7.0–13.0) in the delayed SC1 group ( *P* < 0.01). Overall, we did not identify a statistically significant difference in recurrence rates at SC1 between the standard and delayed SC1 group (standard group 6.4% versus delayed group 6.5%, *P* = 1), even when we extended the timeframe to include only very late SC1 (> 12 months). These very low RRA rates following pEMR with margin ablation of LNPCPs have been consistent across several studies performed in recent years following the randomized trial by the Australian group 12 13 15 17 19 20 . The most recent study by Rex et al, with a median interval of 6.4 months to SC1, also showed a low RRA rate of 4.6% following STSC20.This demonstrates that such results can be reproduced across centers and endoscopists performing high-quality luminal resections, and this is the new benchmark we should be aspiring for.

Importantly, upon convergence of the groups at SC2, no difference in recurrence rate was observed and no advanced lesions (HGD or cancer) were identified among the recurrences. These findings suggest that when performing high-quality pEMR, delaying SC1 beyond 6 months probably does not significantly increase risk of recurrence at this initial surveillance point or risk of developing an advanced lesion within the recurrence. Furthermore, delaying SC1 to 1 year after the index procedure has the advantage of recognizing late recurrence without subjecting the patient to additional colonoscopies.

The current intensive surveillance regimen following pEMR incurs significant burdens for patients and the health care system and may lead to low compliance rates for subsequent colonoscopies. Our data show that most recurrences can be detected during SC1 and that the adjusted OR for recurrence ( Fig. 2 ) does not change significantly between 3 and 18 months. This challenges the existing paradigm and suggests that a more flexible approach to timing of SC1 may be considered without compromising patient outcomes. We believe a more personalized approach based on individual patient characteristics and lesion characteristics is indicated. A good example for this is shown in our analysis of high-risk (high risk for RRA) lesions (i.e those at the ileocecal valve, anorectal junction, fully circumferential lesions, and previously attempted lesions). These lesions, by their nature and location, present unique challenges in terms of endoscopic resection and surveillance and were previously shown to have higher recurrence rates compared with standard LNPCPs 7 11 12 . We also observed higher overall recurrence rates in our cohort for these lesions compared with the general cohort, but we found no significant difference in recurrence rates within this group, between the standard and delayed SC1 groups. This finding suggests that the impact of timing on recurrence should be influenced by lesion characteristics rather than by a strict temporal factor, underscoring the importance of a tailored management strategy for LNPCPs.

Lack of a significant difference in recurrence rates at SC2 between the standard and delayed SC1 groups has potential implications for healthcare resource allocation and patient morbidity associated with additional colonoscopies. Although timing of SC1 did not appear to significantly impact SC2 recurrence rates in our study, it is important to recognize that lesion-specific risk factors, such as size, location, and previous treatment attempts, likely play a significant role in determining risk of recurrence. Therefore, individualized assessment and management based on these factors remains essential.

Our study mirrors previous studies 21 , which noted a higher recurrence rate for large lesions (> 40 mm) at SC1. Our study failed to demonstrate additional risk factors such as intra-procedural bleeding or lesions with high-grade dysplasia at the index colonoscopy ( Table 1 ). Lesion size as a risk factor is well documented as previously shown by the Australian group in the Sydney EMR Recurrence Tool (SERT) 21 , which assigns higher scores to larger lesions. Furthermore, the predictive clinical scoring system SMSA score (size, morphology, site and access), which identifies a subset of patients with a heightened risk of unsuccessful EMR, adverse events, and adenoma recurrence during surveillance colonoscopy, shows that patients with lower SMSA scores exhibited a reduced rate of endoscopic recurrence 22 . Thus, incorporation of predictive tools like SERT and SMSA score into clinical practice can enhance risk stratification and guide individualized surveillance strategies.

Another paramount aspect is adequate EMR training. According to the European Society of Gastrointestinal Endoscopy recommendation, LNPCPs (> 20 mm) should be removed by an appropriately trained and experienced endoscopists, in an appropriately resourced endoscopy center 15 . This will ensure low recurrence rates and enable a more flexible surveillance regimen 23 .

Our findings complement a multicenter randomized controlled trial by Nakajima Takeshi et al., which evaluated surveillance intervals after piecemeal EMR for large colorectal neoplasia 24 . Together, these data support considerations of more flexible, risk-adapted surveillance timing in settings with high-quality pEMR and systematic margin treatment, while recognizing differences in study design and local practice patterns.

Our hospitals are large academic referral centers with a high volume of EMR procedures and our luminal endoscopists had dedicated EMR training and follow the same structured resection technique. This accounts for our low recurrence rates and underscores the importance of a dedicated EMR training programs and use of a meticulous technique.

### Limitations

The retrospective nature of the study introduces inherent biases and generalizability of our findings may be influenced by the specific patient population and healthcare settings.

Although endoscopic scar assessment has been shown to be highly accurate, reliance on endoscopic assessment with biopsy only when recurrence was suspected could miss subtle recurrences, particularly in a low-event setting. Any such misclassification would be expected to bias recurrence estimates toward the null.

Prospective randomized studies with longer follow-up periods could offer a more comprehensive understanding of the impact of delayed surveillance on long-term outcomes.

Although there was a small difference in lesion sizes and locations between the standard and the delayed group, the absolute differences were minor, and we believe these differences do not affect our results and conclusions as seen with the results of PSM.

Reasons for delayed SC1 were not recorded in a fully structured manner for every case, raising the possibility of unmeasured confounding related to access, adherence, and clinical factors.

In addition, we could not reliably distinguish procedures planned as piecemeal from those converted intra-procedurally, nor quantify specific reasons for conversion, which may limit reproducibility across settings.

SC2 completion was low overall and imbalanced between groups (standard 32.7% vs delayed 20.0%), limiting inference on longer-term outcomes. These results are vulnerable to attrition/selection bias. The direction of bias is uncertain: if higher-risk or symptomatic patients preferentially returned for SC2, SC2 recurrence may be overestimated; if healthier or more adherent patients preferentially returned, SC2 recurrence may be underestimated. Accordingly, SC2 findings should be interpreted as exploratory and may not generalize beyond the observed follow-up.

## Conclusions

In this large cohort of LNPCPs resected by pEMR with systematic STSC, delaying SC1 beyond 6 months was not associated with higher recurrence at SC1 and no advanced recurrence was observed at SC1 or SC2. These findings support evaluation of more flexible, risk-adapted surveillance intervals, pending prospective validation.

## Supplementary Material

Supplementary Material Zusatzmaterial
