# Supplementary material for: Delayed surveillance colonoscopy after piecemeal EMR is not associated with increased recurrence rates
Source: Endosc Int Open. 2026 Mar 6;14:a28164998. doi: 10.1055/a-2816-4998 (PMC13063303; doi:10.1055/a-2816-4998)
Supplement: Supplementary file 1 — Supplementary Material [file 10-1055-a-2816-4998_28196679.pdf]

Supplementary Table 1 Surveillance colonoscopy 2 (SC2) outcomes by SC1 status.

SC2 outcome category	n	N	%
Any recurrence detected at SC2	8	167	4.8
SC1-negative → SC2-positive	3	167	1.8
SC1-positive and treated → SC2-positive	5	167	3.0

Denominator: Lesions that completed SC2 (N = 167).
SC1, first surveillance colonoscopy; SC2, second surveillance colonoscopy.
The two SC2-positive subcategories are mutually exclusive and sum to the total SC2 recurrences.
Group-level (standard vs delayed) stratification for these SC2 subcategories is not shown in this table.

Endosc Int Open | DOI 10.1055/a-2816-4998 | © The Author(s).
